# A Sad Reflection

**Published:** 1882-02

**Authors:** 


### A SAD REFLECTION.

One of the heart-sorrows which few parents escape, who live to see their children nearly grown, is the early disposition which both sons and daughters show, to throw off parental control, and exercise their own judgment, in all that pertains to practice and principle.

Youth is vain, hopeful, dogmatic and impatient. At sixteen, seventeen, and even earlier, they have already regarded it as a settled fact that they are largely wiser than those who have gone before. They consider it as a weakness to be pitied, the fears and misgivings which bitter experience have burnt into the father's and mother's heart, and left them all cut and scarred. If the counsels are given in the sternness of parental right, they are met with a feeling which soon grows into defiance; if given with the beseechings of a mother's undying affection, which still clings to a prayer, when command and reason and persuasion, all have failed, they look down on this deep solicitude, this heart-breaking anxiety, with a patronising pityingness, and with silent neglect or compassionate smile, mingled with a feeling amounting almost to contempt for such useless earnestness, and they pass steadily on to courses which, sooner or later, work out their irretrievable ruin.

On a beautiful morning of the past Spring-time, Dr. ——, standing at our door, his head whitening for the grave, and into which he has already passed, said in tones at once earnest, tremulous and deep, "I cannot induce my son to forego the use of tobacco, although he sees in his father its mischievous effects."

That father had long since, with the will as well as with the intellect of a giant, dashed the chain of habit in pieces, doing it the moment he became convinced of the perniciousness of the practice, but not soon enough to have escaped the impress of its disastrous effects, in enfeebled limbs and palsied tremblings, and that too when the hill-top of life had scarcely been reached as to years, but in reality as to him passed a long time ago, one foot being already in the grave, the other on its crumbling verge.

The cruel heedlessness with which the youth of our time pass by the known wishes of their parents, as to what their parents well know would, in good time, add to their comfort, happiness and prosperity, is a sign of the times, and merits a stern rebuke.

Parents may not complain of such neglect; they may not bring it distinctly to the notice of their wayward children, but their hearts are wounded for all that, and many is the tear that is dropped in secret for that self same cause; and the exclamation breaks up from the depth of their affliction: "Is it for this I have suffered and watched, and toiled from their infancy up? Is it for this, I have practiced a life-long self denial, and self sacrifice, and weary wasting labor, until my back is bent with years, my limbs stiffened with work, and my hand hard as bone itself?" and scalding tears flow plenteously down, else the overburdened heart would break in its agony.

Let every child then, having any pretence to heart or manliness or piety, and who is so fortunate as to have a father or mother living, consider it a sacred duty to consult at any reasonable, personal sacrifice, the known wishes of such a parent, until that parent is no more; and our word for it, the recollection of the same through the after pilgrimage of life, will sweeten every sorrow, will brighten every gladness, will sparkle every tear drop with a joy ineffable. But be selfish still, have your own way, consult your own inclinations, yield to the bent of your own desires, regardless of a parent's commands and counsels and beseechings and tears, and as the Lord liveth, your life will be a failure; because "the eye that mocketh at his father, and despiseth to obey his mother, the ravens of the valley shall pick it out, and the young eagles shall eat it."

